# Supplementary material for: Datavzrd: Rapid programming- and maintenance-free interactive visualization and communication of tabular data
Source: PLoS One. 2025 Jul 22;20(7):e0323079. doi: 10.1371/journal.pone.0323079 (PMC12282858; doi:10.1371/journal.pone.0323079)
Supplement: S1 Appendix — We illustrate how Datavzrd supports rich, per-cell visualizations using Vega-Lite and compare its concise, declarative configuration against a minimal Shiny implementation. Example configurations, rendered outputs, and setup instructions are provided for both tools. (PDF) [file pone.0323079.s001.pdf]

Supplement

Custom Plot Example

Datavzrd supports the embedding of fully customized per-cell visualizations using Vega-Lite, enabling rich, declarative plotting beyond the built-in options. This is achieved through the custom-plot configuration, where users can define a function that generates data from the content of a given cell and supply a Vega-Lite specification to render that data. This mechanism allows Datavzrd to visualize arbitrary structured content, including threshold-based markers, score distributions, or even tiny scatter plots directly within table cells. In Figure 1 we show an example used in the Snakemake DNA-seq Varlociraptor workflow.

```
'tumor: observations':
        optional: true
        custom-plot:
          data: |
            function(value) {
                var regex = /([0-9]+)([A|a|R|r]?[E|B|P|S|V|e|b|p|s|v])(\.|[0-9]+)(s|p)(\#|\*|\.)(\+|\-|\*)(\>|\<|\*|\!)(\^|\*)(\$|\.)(\*|\.)/g;
                var effects = {
                    "E": "Equal",
                    "AB": "Barely (Alt)",
                    "AP": "Positive (Alt)",
                    "AS": "Strong (Alt)",
                    "AV": "Very Strong (Alt)",
                    "RE": "Equal (Ref)",
                    "RB": "Barely (Ref)",
                    "RP": "Positive (Ref)",
                    "RS": "Strong (Ref)",
                    "RV": "Very Strong (Ref)"
                };
                var observations = [];
                var orientation = {
                    "*": "unknown",
                    ">": "F1R2",
                    "<": "F2R1",
                    "!": "non standard"
                };

                while ((result = regex.exec(value)) != null) {
                    strand = result[6].replace("*", "±");
                    edit_distance = result[3].replace(".", "0");
                    effect = effects[result[2].toUpperCase()];
                    var quality = "low";
                    if (result[2] == result[2].toUpperCase()) {
                        quality = "high";
                    }
                    observations.push({
                        "strand": strand,
                        "effect": effect,
                        "times": parseFloat(result[1]),
                        "quality": quality,
                        "orientation": orientation[result[7]],
                        "edit distance": edit_distance
                    });
                }
                return observations;
            }
          spec-path: spec_observations.json
        display-mode: detail
```

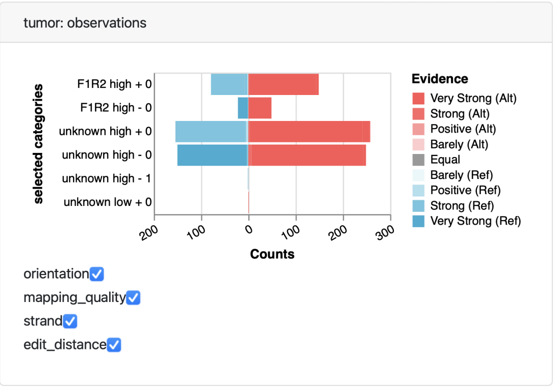

Figure 1 – Custom plot configuration used in https://github.com/snakemake-workflows/dna-seq-varlociraptor. The file content of `spec_observations.json` is available under https://github.com/snakemake-workflows/dna-seq-varlociraptor/blob/master/workflow/resources/datavzrd/spec_observations.json

Shiny vs. Datavzrd

Both Shiny and Datavzrd support the display of interactive tables. Since visually rich, interactive tables are the primary feature of Datavzrd, it provides particularly concise configuration of such tabular views. We illustrate this difference via the following example, with the Shiny implementation (using its Python API) on the left and the Datavzrd configuration for an equivalent table on the right. Note that we restricted the functionality used for this example to the shared features of the two tools. As can be expected, both Datavzrd and Shiny have exclusive features beyond what is used here.

Usage

In the following section we explain the usage of both Shiny and Datavzrd to create the examples below. We assume a properly setup unix based system with a working conda package manager installed.

Shiny

To run Shiny we first create a new Conda environment with all dependencies using `conda create -n shiny-env python=3.12 shiny pandas matplotlib -c conda-forge` and `conda activate shiny-env`. From within the project directory (where `shiny_recreation.py` and `data/anonymized.tsv` reside) we must then launch the app each time we want to inspect the report using `shiny run --reload shiny_recreation.py`. This starts a local web server (by default at `http://localhost:8000`) serving the table (see Figure 2) defined in the python file.

Datavzrd

To create a Datavzrd report we need to install the binary either into the base environment or a dedicated one. The latter is done by running `conda create -c conda-forge -n datavzrd datavzrd`. We then activate the environment via `conda activate datavzrd`. In the directory where the configuration file and `data/anonymized.tsv` are located we then generate the report via `datavzrd datavzrd_config.yaml --output report`. The generated report (see Figure 3) can then be inspected by opening the `index.html` file in the specified `report` output directory.

Configuration

To illustrate the simplicity and efficiency of Datavzrd compared to existing alternatives such as Shiny, we compare two equivalent examples that render a table of genetic variants with categorical color highlighting based on impact and consequence. The Datavzrd configuration (see below) requires only a short YAML file using 28 lines. In contrast, the equivalent Shiny application requires 49 lines of Python code, involving data loading, manual color mapping, style computation, and reactive UI definition.

While the Shiny example uses imperative code with external libraries like pandas, matplotlib, and shiny.express, the Datavzrd approach is entirely declarative. Users define what should be shown, not how it should be computed or rendered. This reduces both the amount of code and the technical expertise needed to create interactive tables with meaningful visual encodings.

Shiny

```python
from pathlib import Path
import matplotlib.pyplot as plt
import pandas as pd
from shiny.express import render, ui

app_dir = Path(__file__).parent
df = pd.read_csv("../data/anonymized.tsv", sep="t")

ui.page_opts(title="Variants in coding regions.", fillable=True)


def impact_to_color(impact):
    impact_color_map = {
        'HIGH': '#ec0000',
        'MODERATE': '#ec5300',
        'LOW': '#ec9b00',
        'MODIFIER': '#ecca00'
    }
    return impact_color_map.get(impact, '#FFFFFF')


def consequence_to_color(consequence):
    category20 = plt.get_cmap('tab20').colors
    unique_consequences = df['consequence'].unique()
      consequence_color_map = {consequence: category20[i % 20] for i, consequence in
enumerate(unique_consequences)}

    def rgb_to_hex(rgb):
       return '#{:02x}{:02x}{:02x}'.format(int(rgb[0] * 255), int(rgb[1] * 255), int(rgb[2]
* 255))

    return rgb_to_hex(consequence_color_map.get(consequence, (1, 1, 1)))


def get_styles(df):
    styles = []
    for idx, row in df.iterrows():
        impact_color = impact_to_color(row['impact'])
        styles.append({
            "rows": [idx],
            "cols": [df.columns.get_loc('impact')],
            "style": {"background-color": impact_color, "color": "white"}
        })

        consequence_color = consequence_to_color(row['consequence'])
        styles.append({
            "rows": [idx],
            "cols": [df.columns.get_loc('consequence')],
            "style": {"background-color": consequence_color, "color": "white"}
        })

    return styles


with ui.card(full_screen=True):
    @render.data_frame
    def summary_statistics():
        styles = get_styles(df)
        return render.DataGrid(df, styles=styles)

ui.include_css(app_dir / "styles.css")
```

Datavzrd

```yaml
name: Variants in coding regions.
datasets:
  sample-coding:
    path: data/anonymized.tsv
    separator: "t"
views:
  sample-coding:
    dataset: sample-coding
    render-table:
      columns:
        impact:
          plot:
            heatmap:
              scale: ordinal
              domain:
              - HIGH
              - MODERATE
              - LOW
              - MODIFIER
              range:
              - '#ec0000'
              - '#ec5300'
              - '#ec9b00'
              - '#ecca00'
        consequence:
          plot:
            heatmap:
              type: nominal
```

Below, the resulting rendered views for the Shiny and Datavzrd case can be seen (while hiding columns that exceed the page width). The full Datavzrd with all exclusive features is available under https://datavzrd.github.io/example-molecular-tumor-board.

Shiny result

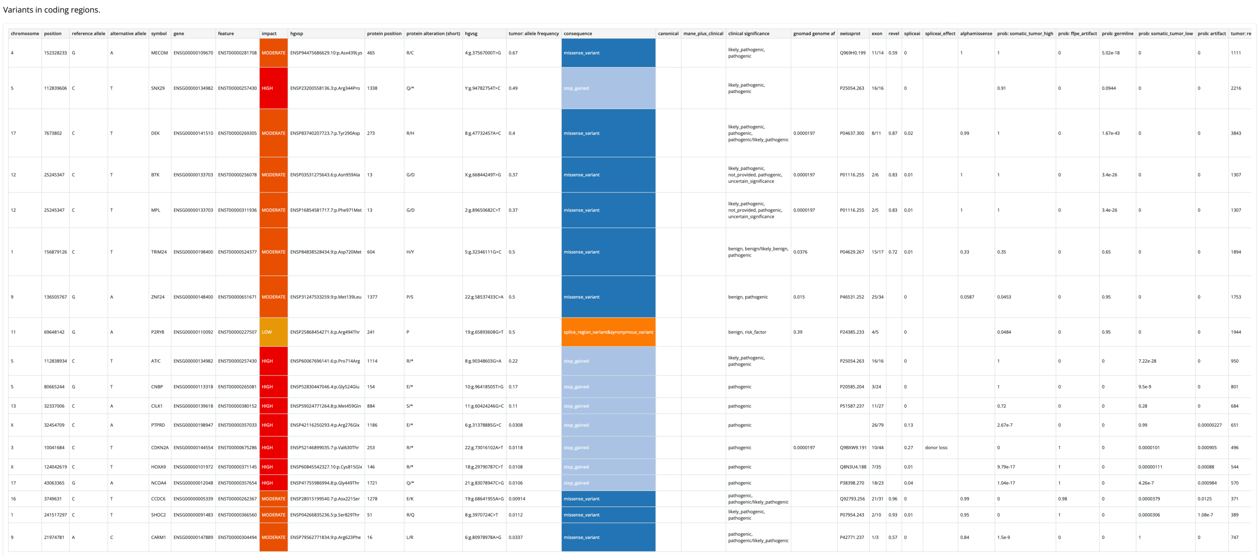

Figure 2 – Shiny Example

Datavzrd result

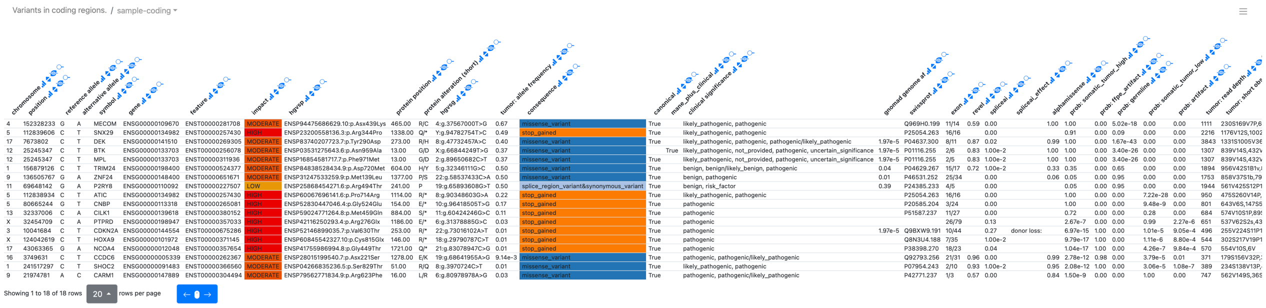

Figure 3 – Datavzrd Example
