# Supplementary material for: Datavzrd: Rapid programming- and maintenance-free interactive visualization and communication of tabular data
Source: PLoS One. 2025 Jul 22;20(7):e0323079. doi: 10.1371/journal.pone.0323079 (PMC12282858; doi:10.1371/journal.pone.0323079)
Supplement: S2 File — Interactive Datavzrd report showcasing genomic variants with associated scores and predictions in a molecular tumor board context. The dataset has been de-identified by altering gene names and coordinates; see the original workflow at https://github.com/snakemake-workflows/dna-seq-varlociraptor and explore the interactive report at https://datavzrd.github.io/example-molecular-tumor-board. (ZIP) [file pone.0323079.s002.zip › datavzrd-report/sample-noncoding/index_1.html]

datavzrd report


1. Variant calls somatic\_or\_germline\_pathogenic\_risk\_factor\_drug\_response
2. sample-coding
   sample-noncoding

#### No data

datavzrd

- 2.44.2
- github

created Wed Nov 6 23:05:43 2024
