# Supplementary material for: Datavzrd: Rapid programming- and maintenance-free interactive visualization and communication of tabular data
Source: PLoS One. 2025 Jul 22;20(7):e0323079. doi: 10.1371/journal.pone.0323079 (PMC12282858; doi:10.1371/journal.pone.0323079)
Supplement: Attachment — Submitted filename: Response_to_reviewers_Datavzrd.pdf [file pone.0323079.s003.pdf]

Response to reviewers: Rapid programming- and maintenance-free interactive visualization and communication of tabular data

June 26, 2025

We thank both reviewers for the comprehensive and positive reviews. In the following, we answer each comment in detail.

Reviewer 1

General comments The paper presents a framework called Datavzrd that allows easy visualization of tabular data via HTML pages. It does not report substantial research findings, but presents a new research method which could be interesting for many disciplines dealing with tabular data. The paper is written in comprehensible language and presented in an appealing style. It claims many advantages of their tool over existing alternatives, but misses evidence for these claims.

Response We thank the reviewer for the constructive summary and for recognizing the potential interdisciplinary relevance and clarity of the manuscript. We have reworked various areas of the manuscript to better and more clearly support the claimed advantages. See our responses to specific comments below.

Comment 1 The authors claim that their tool allows more efficient visualizations with less code than existing alternatives, in particular Shiny. This is argued in text, but not substantiated by examples. An example workflow for a simple data table should be included, which puts the Datavzrd approach in contrast to Shiny (similar to the supplementary material plus brief explanations).

Response We have extended the comparison in the supplement with explanations on the usage of both Shiny and Datavzrd. Additionally we have added another new supplementary section that further explains the comparison of the Shiny and Datavzrd configuration example. The supplement highlights that the Datavzrd approach requires about half of the lines compared to Shiny (28 vs. 49), involves no programming, and avoids data manipulation or plotting libraries. Instead, the full configuration is expressed in a short, human-readable YAML file. This addition should further substantiate our claim that Datavzrd enables more concise and accessible visualizations compared to imperative frameworks like Shiny.

Comment 2 In Sec. 1 Excel and the xlsx format are mentioned. It is not clear, however, which other – esp. non-proprietary – file formats are supported and how the tool is applied to these.

Response We have clarified the supported file formats in the revised manuscript. In short, Datavzrd supports CSV (and TSV) or Apache Parquet[1] as input formats, both being non-proprietary. For exporting tabular views from the browser display, Datavzrd supports CSV and Excel.

Comment 3 Furthermore it remains unclear how the Datavzrd definition of visualizations interacts with the dataset. There are very few code snippets (e.g. Fig. 4) which, however, do not show how visualizations are defined. An example workflow as suggested in comment 1 could help.

Response We have unified the examples of column configurations into a single figure and extended them with a bar and pill plot definition. Furthermore we expanded the supplement with an example of a custom plot definition that uses Vega-Lite syntax. In short, one can say that the user specifies a Datavzrd configuration file. When applying that file to one or more tables (by invoking Datavzrd as a command line tool), Datavzrd uses the specified visualizations to render the tables into self-contained interactive HTML which can then be send around, stored in some cloud, be attached as a supplementary to a manuscript, or hosted via static web servers (including e.g. github pages). To clarify the core workflow, we expanded the manuscript with an explanation on how configuration and dataset interact:

Using simple YAML-based declarative specifications, users define datasets along with the desired visualizations for each column. The self-contained report is then generated by executing Datavzrd with the command `datavzrd path/to/config.yaml -o path/to/output`.

[1] `https://parquet.apache.org/`

Comment 4 In the related works section of the introduction, D3, Plotly, and Dash could be mentioned as further widely used tools (also requiring lots of code, though).

Response We thank the reviewer for the suggestion. In response, we have added a sentence to the related works section of the introduction mentioning Dash, D3.js, and Plotly, and positioning them in the broader spectrum of solutions:

Similar to Shiny, Dash allows to create interactive dashboards with Python code, but also requiring a running server process. [...] Of course, charting libraries such as D3.js or Plotly enable the creation of interactive visualizations, but using them within tables requires substantial bespoke coding and web-development effort.

Furthermore we have clarified the position of Vega-Lite, Vega and D3.js within Datavzrds ecosystem in the interactivity section:

Datavzrd relies on Vega-Lite as its underlying engine for generating a wide variety of plots. Internally, Vega-Lite specifications compile to Vega, which itself builds on D3.js for rendering. Datavzrd provides an even higher level of abstraction with its available pre-made plot configurations for `ticks`, `bars`, `pills` and `heatmaps`. When specified for a column (cf. Figure 9), these visualizations are automatically applied and configured to every cell within that column.

Comment 5 Figure 2 is too small and thus unreadable. It should be rotated by 90 degrees to fill a whole page.

Response We have rotated the figure accordingly to improve readability.

Comment 6 Code and visualization in Fig. 4 do not match. Furthermore, the very small code snippet does not explain what a "spell" is and how the values true and false are translated in visual representations.

Response We fully agree with the reviewer. In response, we have switched Figs. 4 and 5 to better align the code with the corresponding visualization. We also improved the explanation of how the spell definition on the left corresponds to the rendered report example on the right.

Comment 7 Several references are missing bibliographical information such as journal/conference, page numbers etc., such as [14], [16], [17], [22]. Furthermore, journal names should be capitalized. More care should be taken when preparing the next version.

Response We thank the reviewer for pointing out the missing details in some of the references. We have carefully reviewed all references in the manuscript and, to the best of our ability, added all available bibliographic information, including journal/conference, DOIs, and page numbers where applicable.

For the specific reference mentioned by the reviewer, we have updated the entry to include additional details. A summary of the changes for the specifically mentioned references is provided in the list below.

14 *A multi-analytical study of [...]* We have added more information including the article identifier **eadp1917** since *Science Advances* doesn't use traditional page numbers.

16 *Sustainable data analysis with Snakemake* We have added the missing DOI and issue number. F1000 does not provide page numbers.

17 *The Not-So-Same-Origin Policy* We have added a missing author as well as a link to article.

22 *Zenodo* We could not find a better option of citing *Zenodo* themselves. We use the BibTex entry they provide to cite them at the bottom of their "About" page: `https://about.zenodo.org`.

Reviewer 2

General comments In this paper, the authors introduce Datavzrd, a framework designed for generating server-free, portable, and interactive visual reports from tabular data. The system leverages a declarative specification language to facilitate rapid report configuration, eliminating the need for imperative programming. The paper is generally well-written and is supported by illustrative examples and clear explanations.

Response We thank the reviewer for the positive and encouraging feedback. We are glad the manuscript provided clear explanations and illustrative examples that were helpful in conveying the core ideas and design of Datavzrd.

Comment 1 One notable advantage the authors might emphasize is that client-side JavaScript frameworks reduce server load, as all rendering, interactivity, and event handling occur in the client's browser. Given the capabilities of modern PCs and smartphones, this approach is both practical and scalable.

Response We thank the reviewer for this helpful suggestion. We have incorporated this point into the manuscript:

Additionally, it also eliminates server maintenance and reduces server load by shifting processing load to the browser (without hampering scalability, see section Scalability).

Comment 2 However, to substantiate the authors' claim regarding the uniqueness of Datavzrd, it is important to include a more comprehensive comparison with existing client-side JavaScript frameworks. Specifically, a discussion of alternatives such as D3.js, Vega, Plotly.js, and even older technologies like XSLT would strengthen the paper by placing Datavzrd within the context of existing tools.

Response We have extended our discussion of Datavzrd's functionality in the context of other approaches in the introduction (thereby also adding XSLT, Dash, D3.js, Vega, Plotly.js):

Naturally, any general purpose programming language (like e.g. Python or R) or transformation languages (e.g. XSLT) could be used (also e.g. in comparison with helper libraries like great-tables) to implement entirely custom reporting. [...] Similar to Shiny, Dash allows to create interactive dashboards with Python code, but also requiring a running server process. [...] Of course, charting libraries such as D3.js or Plotly enable the creation of interactive visualizations, but using them within tables requires substantial bespoke coding and web-development effort.

Furthermore we have clarified the position of Vega-Lite, Vega and D3.js within Datavzrds ecosystem in the interactivity section:

Datavzrd relies on Vega-Lite as its underlying engine for generating a wide variety of plots. Internally, Vega-Lite specifications compile to Vega, which itself builds on D3.js for rendering. Datavzrd provides an even higher level of abstraction with its available pre-made plot configurations for `ticks`, `bars`, `pills` and `heatmaps`.

Comment 3 Many server-based reporting tools provide advanced aggregation capabilities such as window functions, multi-level aggregations, predictive analytics, and pivot operations. These typically require multiple data passes and significant memory. The authors should clarify the scope of Datavzrd's aggregation capabilities, which currently seem limited to simple aggregations, such as column histograms and heatmaps.

Response We clarified the limit of Datavzrd's aggregation capabilities together with an explanation on why these are not developed further at the moment:

Currently, Datavzrd's aggregation capabilities are limited to simple, per-column summaries such as histograms. More complex operations like window functions or pivot tables are not supported. We see this as a deliberate design choice: such aggregations are

better handled upstream in the analysis workflow, where they can be executed transparently and reproducibly. In future versions, we plan to extend Datavzrd with lightweight interactive features such as scatterplots for user-selected column pairs.

Comment 4 On page 5, the authors state: "Via compression methods and data partitioning strategies, Datavzrd is scalable towards big datasets without overwhelming the browser memory while still maintaining interactive capabilities." The authors should quantify performance characteristics to improve clarity and reproducibility. Consider including details such as: Performance benchmarks: e.g., "Interactive operations such as filtering respond in under 300 milliseconds with datasets up to 5 GB in size." Test environment: e.g., "Tested on a machine with 32 GB RAM, Intel i7 CPU, running Windows 11." Visualization types supported: e.g., "Line chart, heatmap, 3D surface plot." Resource usage: e.g., "CPU and RAM utilization during peak operations." Avoid vague claims such as "handles big files efficiently" without accompanying empirical evidence.

Response We conducted additional benchmarking experiments on a large synthetic dataset and added the resulting quantitative performance characteristics for filter operation times and the corresponding hardware and software specifications to the manuscript.

For a synthetic dataset with 100,000 rows and 30 columns (including nominal, integer, and string-valued columns), interactive filtering operations consistently complete in under 100 ms. Measurements were taken using the browser's `performance.now()` API in Safari 18.5 on macOS 13.7.6 with a 3.8 GHz Quad-Core Intel Core i5 and 48 GB of RAM.

Regarding supported visualization types, we clarified that, in addition to Datavzrd's predefined plot types (see Section "Feature overview" incl. new figure), users can embed fully custom Vega-Lite specifications. This enables the creation of a wide range of visualization types supported by Vega-Lite, including scatter plots, maps, faceted views, and more.

Through this mechanism, users can harness the full expressiveness of Vega-Lite, which supports a wide range of visualizations such as scatter plots, line and area charts, bar charts, heatmaps, geographic maps and faceted layouts (see `https://vega.github.io/vega-lite/examples/` for the extensive set of supported types).

Comment 5 Figure 2 is too small to be legible. The authors should enlarge the figure to ensure that the visualizations are clearly discernible.

Response We have rotated the figure to increase the size and readability.

Comment 6 The manuscript states: "Datavzrd provides a publish subcommand, which automates the upload of reports to a newly created or existing GitHub repository and enables hosting via GitHub Pages with a single click. The phrase "with a single click" is misleading. A more accurate description would be: "...and enables hosting via GitHub Pages using a simple command-line invocation." The process requires executing a command in the following format: `datavzrd publish --repo-name --report-path [--org ]` This is not a one-click operation and should be described as such.

Response This has been indeed phrased very badly and is misleading. The click operation is needed afterwards to enable GitHub pages for the repository since this is not possible via the GitHub CLI. We have clarified the section of the manuscript accordingly:

For enhanced accessibility and ease of hosting, Datavzrd provides a `publish` subcommand, which automates the upload of reports to a newly created or existing GitHub repository using a simple command-line invocation. Afterwards it provides the user with a link to the repository settings where GitHub Pages need to be enabled manually — once upon initial publication of the repository.

Comment 7 The authors note: "Datavzrd stores data in JavaScript files, which are then loaded via static script tags." This is an important implementation detail. However, the authors should also acknowledge that when data updates are needed, the visualizations must be regenerated using the Datavzrd command line. This introduces some operational overhead, and its mention would provide a more balanced and transparent evaluation.

Response We have clarified in the manuscript that updating the input dataset requires regenerating the report:

This approach requires regenerating the report whenever the input dataset changes; however, because data and configuration are completely separated, no updates to the latter are needed as long as the structure of the data remains unchanged. We recommend embedding the invocation of Datavzrd into automated workflows (e.g., using Snakemake, Nextflow , or CWL) to facilitate reproducible report generation after data updates.

Comment 8 The statement: "For the first time, this enables the collaborators and readers to interactively explore the data underlying the findings..." is overly assertive. Without rigorous comparative analysis, claims of precedence should be avoided.

Response We agree and have removed the phrase "For the first time".

Comment 9 Live Demo: `https://datavzrd.github.io/example-report-moscot/Suppl.%20Tab.%2013/index_1.html` The Birth date field is interpreted as a string, preventing effective range-based searches. Additionally, the column histogram is not time-ordered. These are areas where usability could be improved.

Response We agree that usability can be improved by supporting more specific data types such as temporal data. We have added a corresponding note to the Outlook section of the manuscript and created a GitHub issue to track this enhancement: `https://github.com/datavzrd/datavzrd/issues/968`.

Furthermore, we plan to extend support to additional data types, such as temporal data.

Comment 10 `https://datavzrd.github.io/example-report-moscot/Suppl.%20Tab.%207/index_1.html` In the third field, after initiating a search, the data range in the histogram appears confusing. This could hinder interpretability and should be refined.

Response We have addressed this issue in `https://github.com/datavzrd/datavzrd/commit/df88962a24eca62bed945ee3201a4d2953b6c6ed` by using scientific notation for the axis labels of the brush plot. The corresponding report (as well as all others) are also updated using the latest version of Datavzrd.

Comment 11 GitPod Tutorial The tutorial is well-structured and user-friendly. However, a bug exists: after executing the show-report-url command and clicking the generated link, the hide button on top of each field does not actually hide the corresponding field in the report. This issue should be addressed.

Response Thank you for pointing this out. We have fixed the issue in `https://github.com/datavzrd/datavzrd/commit/5ce8fd01b8e8de908e4a72a31bc13327dfb68e79`.

Comment 12 GitHub Live Preview `https://datavzrd.github.io/datavzrd/index.html` The font size in the Markdown file is inconsistent and should be normalized for a professional appearance.

Response We have addressed the issue by normalizing the appearance of Markdown heading levels. Specifically, we remapped Markdown headings to use consistent font weights (e.g., `h1` → weight 900, `h2` → weight 800) instead of varying font sizes. This change ensures a more professional and coherent visual hierarchy. The corresponding changes are available here:

`https://github.com/datavzrd/datavzrd/commit/29dbb42f65cdcd4f8175689e88b6fe34acae3ba0`.

Comment 10 The hide button on top of each field does not actually hide the corresponding field in the following links: `https://datavzrd.github.io/datavzrd/oscars/index_1.html` `https://datavzrd.github.io/datavzrd/movies/index_1.html`

Response We have fixed the issue in `https://github.com/datavzrd/datavzrd/commit/5ce8fd01b8e8de908e4a72a31bc13327dfb68e79`.

Comment 10 Additionally, data visualizations are missing in the following links: `https://datavzrd.github.io/datavzrd/movies-plot/index_1.html` `https://datavzrd.github.io/datavzrd/oscar-plot/index_1.html`

Response We have fixed the issue in `https://github.com/datavzrd/datavzrd/commit/24c99f064f0a49a628403301171d28e836645edb`.

Editor

General comments The paper introduces Datavzrd, a declarative framework for generating server-free, interactive HTML visualizations from tabular data. It is clearly written and presents an appealing tool that could benefit a wide range of disciplines working with structured data. The framework emphasizes ease of use, portability, and client-side rendering, reducing the need for complex server infrastructure or imperative programming.

Response We thank the editor for the positive summary and for highlighting the strengths of Datavzrd in terms of usability, portability, and especially interdisciplinary relevance. We are pleased that the manuscript communicates these aspects clearly.

Comment 2 However, the manuscript lacks detailed workflows, comprehensive comparisons with existing tools (e.g., D3, Plotly, Dash), and quantified performance evaluations. Technical details, including supported data formats and visualization configurations, are either missing or insufficiently explained. Visual elements like figures and code snippets also need improvement for clarity and completeness.

Response We thank the editor for this helpful summary. We have addressed all these points in detail in response to the reviewers' comments. Specifically:

- We added an extended example workflow and a side-by-side comparison with Shiny (Reviewer 1, Comment 1).
- We clarified and expanded the discussion of related tools including D3, Plotly, and Dash (Reviewer 1, Comment 4; Reviewer 2, Comment 2).
- We provided quantitative performance benchmarks, hardware specifications, and clarified supported plot types and file formats (Reviewer 2, Comment 4).
- We improved figures and added clearer, more complete code snippets (Reviewer 1, Comments 3, 5 and 6).

Comment 3 The authors should ensure that the supporting files (such as datavzrd-supplement.pdf and datavzrd-report-supplement.zip and its contents) are accessible in the manuscript's Supporting Information section so they are not missed by the reviewers and are eventually accessible to the readers of the publication as well.

Response We thank the editor for the suggestion. We verified that the file datavzrd-report-supplement.zip opens correctly across different systems and that its contents are accessible without issue (download, unzip, open index.html). To further facilitate easy access, we also published the report online via GitHub Pages using `datavzrd publish` to `https://datavzrd.github.io/example-molecular-tumor-board`.

Comment 4 Overall, Datavzrd shows good potential, but the paper would benefit from additional empirical support, clearer technical exposition, and a more balanced discussion of its limitations and place within the broader visualization tool landscape.

Response We thank the editor for this constructive summary. In revising the manuscript, we carefully addressed the points raised by both reviewers, including the need for empirical benchmarks, clarifictaion of technical aspects, and a more balanced discussion of limitations and related tools.
